# A Case Study in Serendipity: Environmental Researchers Use of Traditional and Social Media for Dissemination

**DOI:** 10.1371/journal.pone.0084339

**Published:** 2013-12-13

**Authors:** Clare Wilkinson, Emma Weitkamp

**Affiliations:** Science Communication Unit, University of the West of England, Bristol, United Kingdom; Universidade de Brasília, Brazil

## Abstract

In the face of demands for researchers to engage more actively with a wider range of publics and to capture different kinds of research impacts and engagements, we explored the ways a small number of environmental researchers use traditional and social media to disseminate research. A questionnaire was developed to investigate the impact of different media as a tool to broker contact between researchers and a variety of different stakeholders (for example, publics, other researchers, policymakers, journalists) as well as how researchers perceive that their use of these media has changed over the past five years. The questionnaire was sent to 504 researchers whose work had featured in a policy-oriented e-news service. 149 valid responses were received (29%). Coverage in traditional media (newspapers, broadcast) not only brokers contact with other journalists, but is a good source of contact from other researchers (n=47, 62%) and members of the public (n=36, 26%). Although the use of social media was limited amongst our sample, it did broker contact with other researchers (n=17, 47%) and the public (n=10, 28%). Nevertheless, few environmental researchers were actively using social media to disseminate their research findings, with many continuing to rely on academic journals and face-to-face communication to reach both academic and public audiences.

## Introduction

Research communication and the impact of new technologies are changing the way research conclusions are communicated, at the same time as demands for greater access to information are increasing [1]. In fact Peters [2] argues that “the practice of public communication seems to be changing from an exceptional activity requiring justification to a default activity that is accepted as an integral part of the research process” (p. 219). Researchers can use a variety of strategies to disseminate the outputs of their research, as well as traditional media such as newspapers, radio and television, emerging formats include open access academic journals, disciplinary repositories, institutional repositories and personal websites [3]. In addition social media, provide a variety of opportunities to promote and disseminate the latest research [3]. 

Science for Environment Policy (SfEP) is an e-news service designed to facilitate the transfer of scientific knowledge to the policy community. Managed by the Science Communication Unit since 2006 [4], the service is funded by the European Commission. It should be acknowledged that the co-authors of this paper have both had involvement in this service as an internal evaluator and editor respectively. There is no cost to the researchers whose work is featured nor to the policymakers, business leaders and researchers who subscribe to the service. Research featured by the service is selected using traditional news values: relevance to the policy community, magnitude of impact (particularly impact across Europe), surprise (unexpected findings), good or bad news, quality of research (judged partly through peer review, but also through internal review within the project team) [5]. As such, one can assume that this research may have wider appeal to institutional actors (e.g. in national/local policy arenas) and the broader media. Furthermore, the first step in translating the research from the rarefied language of academia into language suited to a policy community (which, admittedly, is still more complex than one finds in a national newspaper) has been taken. With this in mind, in 2010 a policy was implemented of encouraging researchers to make further use of the materials produced. All researchers featured in the news service are sent an email together with a copy of the final article produced and encouraged to use this for further dissemination activities. Researchers are asked to credit the news service as the original source, should they use the article directly (e.g. in a blog). 

Recently, we have explored with these researchers what, if anything, they do with the materials that are sent to them and at the same time we have investigated how their use of broader dissemination tools has changed over the past five years, as well as the types of outcomes they have noted from communicating about their research.

### Context of dissemination

In recent years there has been a growing emphasis world-wide on encouraging scientists to communicate research findings to publics outside the traditional academic communities. Many grant funding agencies (e.g. the National Science Foundation in the US and all Research Councils in the UK) now require grant applicants to provide a dissemination plan that includes communication with non-academic groups, be they policymakers, potential research beneficiaries or the wider public community [6]. Russell et al. [7] goes further, recommending that all scientists proactively engage with communications professionals and the media to communicate their work in a more open manner. Peters et al. [8] (p. 204) report that in their survey of scientists in five countries over two thirds of scientists had interacted with the media in the past three years. Nevertheless, “many scientists indicated that they felt uncertain and perceived a lack of control” in their interactions with the media [8]. 

Despite encouragement to communicate research, there can be a perception that scientists remain reluctant to seek media attention, and that this can be influenced by their perceptions of the handling of past scientific controversies [9]. Some scientists operate with minimal understandings of the science mediation process and their own role within it [10]. Although it is increasingly estimated that stories are packaged and prepared for media coverage by the scientific community [11,12], individual scientists may fail to engage proactively with communications professionals, such as university press officers [13]. Khot [13] suggests that scientists may simply forget to alert press officers to their research or to consider approaching traditional media as a tool for communicating their research.

Traditional media outlets, are, of course, not the only outlet that researchers could use to disseminate their research findings and indeed research in the US suggests that amongst younger age groups, internet sources are preferred to traditional media as sources of information [14]. The internet offers a wealth of opportunities for public communication, many of which allow users high control of message fidelity and so might appeal to the media shy scientist who would otherwise be wary of a journalist misinterpreting their research. Van Eperen and Marincola [15] quote a survey suggesting that scientists are active participants in digital life, with 50% of respondents believing that blogs, discussion groups, online communities and social networking are beneficial to sharing ideas with colleagues. While Holliman [16] (p. 841) argues that “the shift towards a digital, globalised media landscape affords greater levels of interaction and participation to those with access to the web and the skills to produce, distribute, share, archive and retrieve scientific information.” Though this includes scientists, Trench [17] argues that with a few exceptions scientific blogs, for example, have not yet lived up to their potential as a tool to encourage both scientist to scientist and scientist to public communication. More recent research suggests scientists are more likely to use traditional media sources in their own information seeking, but perceive sources such as blogs and social networking to have strong social and political influences [18].

Nisbet and Scheufele [19] highlight the need to take a broad approach to science communication to ensure that scientific information is available to a wide range of audiences, not just those elite audiences who may already have an interest in science. This includes a focus on traditional media, particularly local media and web platforms that may reach non-traditional science audiences. However, this should not be seen as a ‘re-branding’ exercise or continuation of the deficit model, where the public is seen as an empty vessel to be filled with information, but an opportunity to develop meaningful relationships with media and wider publics. As Nisbet and Scheufele [19] (p. 1776) warn, “anytime public engagement is defined, perceived, and implemented as a top-down persuasion campaign, then public trust is put at risk.” Such scholars therefore caution approaches which could be perceived as simply public relations exercises. 

Within this framework, this research explored the ways that researchers whose work has featured in one news service use the opportunities afforded by the internet, social and traditional media to disseminate their research findings, how these have changed and whether they make further use of materials provided by the news service. 

## Materials and Methods

Researchers whose work featured in Science for Environment Policy (n=504) were invited by email to participate in an online survey. This comprised researchers whose academic journal papers had been the subject of a news article or a report between January 2011 and June 2012. The request was sent to the corresponding or first author of the academic paper. It should be acknowledged that prior participation in SfEP could suggest greater receptivity amongst our respondents to media coverage of their work. However it is also important to highlight that researchers do not agree to coverage in SfEP in advance, nor do they volunteer their work for inclusion. Two distributions of the survey were sent, and 77 automatic replies were returned as undeliverable or out of office over the period of the survey. The research had ethical approval from the University of the West of England, Bristol Research and Governance system. Consent was confirmed via electronic completion of the questionnaire. 

149 valid responses were received, with no incomplete responses, giving an overall response rate to this survey of 29%. Analysis of the data occurred using SPSS. It should be noted that response rates to individual questions varied, for example 76 respondents answered questions around traditional media coverage, compared to 36 for social media and therefore percentage response rates show variation amongst individual questions. 

The majority of respondents (82%, n=122) worked in academia or research institutions and 80% (n=119) worked in EU countries. Of this, the biggest single group (15%) came from the UK, and another large group (13%) from Spain. Of the 30 (20%) researchers from non-EU countries, 20 were from the USA. 

## Results

### Impact of media coverage

**Figure 1:**
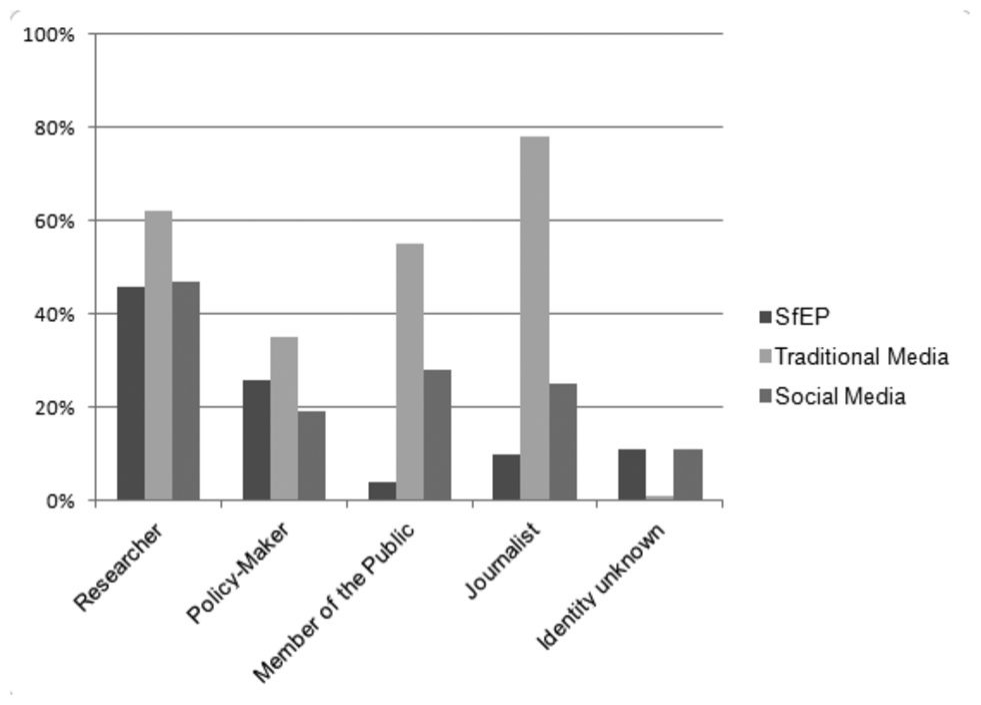
Contact as a result of research featuring in different media. Different media options included Science for Environment Policy, traditional media and social media. Respondents could select more than one category.

Researchers were asked about the impact, in terms of contact from different groups, of their research being featured in the policy focused news alert, traditional media (print and broadcast media) or social media (Figure 1). Overall, 56% (n=84) of researchers featured in SfEP had been contacted by someone, compared with 93% who had coverage in traditional media (it should be noted that this percentage was influenced by the high degree of journalistic contact) and 72% (n=26) who had experienced coverage in social media. Traditional media remain an excellent source of further contact with others. However, when contact was explored in detail, the data suggest that social media and specialist news services such as SfEP are also modestly emerging as methods for promoting contact. 

Not surprisingly, being featured in Science for Environment Policy is likely to result in contact from policymakers, a quarter of respondents had been contacted by this group (n=36, 26%) or researchers (n=65, 46%), reflecting the readership of the publication. The traditional media, in addition to the contact it brokers with journalists, is also a good source for contact with other researchers (n=47, 62%), as well as more predictably members of the public (n=42, 55%). This of course, does not mean that social media has no effect, and the small number of respondents that were using social media to disseminate their work suggested some clear outcomes. Almost half (n=17, 47%) that were using social media had been in contact with other researchers, and 28% (n=10) had been in contact with a member of the public via that route. 

Within this limited dataset there were no clear trends regarding use of traditional and social media, related to the type of institution (e.g. EU, academia, industry etc.) where the researcher worked. Similarly only limited data could be drawn out around specific country and likelihood to use traditional and social media. However when country based data were re-coded to take a regional perspective, two key observations were made (Table 1). Firstly, researchers in Northern Europe appeared most likely to be ‘seeking’ traditional media coverage, 61% (n=22) of researchers from Northern Europe sought coverage of this type. From a social media perspective however it was the Southern and Northern European countries that were more dominant, with just over 20% of researchers seeking such dissemination. Western European researchers appeared to place less focus on seeking dissemination, with just over half (n=19) of researchers based in these countries seeking traditional media coverage, and 16% (n=6) actively using social media. Though it would be interesting to examine trends in Eastern Europe, the minimal data collected from these countries limited this option. 

**Table 1:** Researcher Region seeking traditional/social media coverage.

European and ‘Other’ Countries by Region*	Traditional Media	Social Media
**Western Europe**		
Includes; Austria, Belgium, Germany, France, Luxembourg, Netherlands (n=37)	19 (51%)	6 (16%)
**Eastern Europe**		
Includes; Bulgaria, Czech Republic, Hungary, Poland, Slovakia, Romania (n=3)	1 (33%)	0 (0%)
**Southern Europe**		
Includes; Cyprus, Italy, Malta, Greece, Portugal, Slovenia, Spain (n=39)	15 (38%)	8 (20%)
**Northern Europe**		
Includes; Denmark, Estonia, Finland, Ireland, Latvia, Lithuania, Sweden, United Kingdom (n=40)	24 (60%)	9 (22%)
**Other**		
Includes; United States, Australia, Canada etc. (n=30)	21 (57%)	8 (22%)

^*^ Regional categorisation based on UN Geoscheme for Europe

We explored the types of contact promoted by different types of media (Table 2), to understand the nature of these impacts. 63 respondents, just over 40%, recorded a more specific outcome from their work featuring in SfEP. The most likely outcome, at almost half of the respondents who had experienced further impacts, was discussion of research with other researchers (n=31), however it was also notable that 44% (n=28) had seen the reporting picked up via social media. It is likely that some of this social media comment would be traced back to activities undertaken by SfEP (e.g. @SfEP Twitter feed), but is noteworthy nonetheless. In addition, in contrast to the traditional and social media, coverage in SfEP appeared to be generating a diversity of outcomes, in that 19% (n=12) reported other outcomes in the additional comments section (e.g. mentioned in Departmental News, formed the basis of a press release, generated contact for media coverage). This suggests that whilst SfEP is having a relatively similar impact to more traditional media routes, there are perhaps other aspects of diversity in terms of the types of interest that coverage is generating. 

**Table 2:** Other outcomes as a result of research featuring in different media.

Other outcomes**	SfEP	Traditional Media	Social Media
I have been invited to participate in a conference	17 (27%)	38 (57%)	10 (45%)
I have discussed my research with policy-makers	19 (30%)	32 (48%)	7 (32%)
I have discussed my research with other researchers	31 (49%)	41 (61%)	8 (36%)
I have discussed my work with members of the public	11 (17%)	42 (63%)	8 (36%)
I have been invited to write an article for a newspaper or magazine	11 (17%)	32 (48%)	17 (77%)
I have been invited to write an article for a website	4 (6%)	27 (40%)	8 (36%)
My research was mentioned in social media	28 (44%)	39 (58%)	12 (54%)
Other	12 (19%)	2 (3%)	0 (0%)
Total	63	67	22

^**^ Respondents could select more than one category.

67 respondents, 88% of those whose work had featured in the traditional media, reported additional outcomes. In general, levels of outcome were high via traditional media routes, with around half of respondents reporting outcomes across most of the categories. As might be anticipated the traditional media routes remained high in attracting public attention at 63% (n=42), closely followed by prompting discussion of the research with other researchers (n=41, 61%). There was also a sense that featuring in the traditional media is further disseminated via social media routes, as over half (n=39, 58%) of the survey respondents had seen their work referred to in social media after it had featured in traditional media coverage.

In terms of dissemination that first appeared within social media, 22 respondents discussed further outcomes from that setting. Here the most notable aspect related to its potential for further dissemination, 77% (n=17) reported that it had led to an invite to write for a newspaper or magazine, far higher than this outcome via SfEP or the traditional media route, and similarly there was a good likelihood that other social media would refer to the research (n=12, 54%). 

### Reuse of news alert materials

Researchers whose work is featured in Science for Environment Policy are invited to make further use of the news alert articles produced by the service. On publication in the service, researchers are sent a copy of the published article, a link to its location on the news service website and an invitation to further distribute or otherwise reuse the material. 

Overall, 30% (n=44) of researchers reported reusing information produced by the news alert service for dissemination purposes. We asked these researchers to give examples of the ways they were reusing material. A range of ‘repurposing’ of information was undertaken by the researchers responding to the survey. For example, researchers had used the information in presentations (n=4), to support a funding bid (n=2), posted it on social media (n=2), used it in press releases and printed and distributed details at a public meeting (n=1). One researcher reported using the information in an impact statement, either as part of the research excellence framework assessment undertaken in the UK or in an application for research council funding. In addition, researchers had distributed the content to people outside their institution (n=16). 

When we compared the researchers further disseminating their information, with those that were also seeking traditional and social media coverage, there were only moderate trends. 62% (n=26) of those further disseminating their SfEP coverage were also seeking traditional media coverage, and 28% (n=12) disseminating SfEP work were also pursuing social media dissemination. This would suggest that it is not necessarily the case that ‘active’ disseminators are mapped across all three categories, and that researchers may be selective or limited in the opportunities they pursue. However, as the numbers indicate this was only a moderate amount of re-purposing overall.

### Changes in dissemination routes

**Figure 2:**
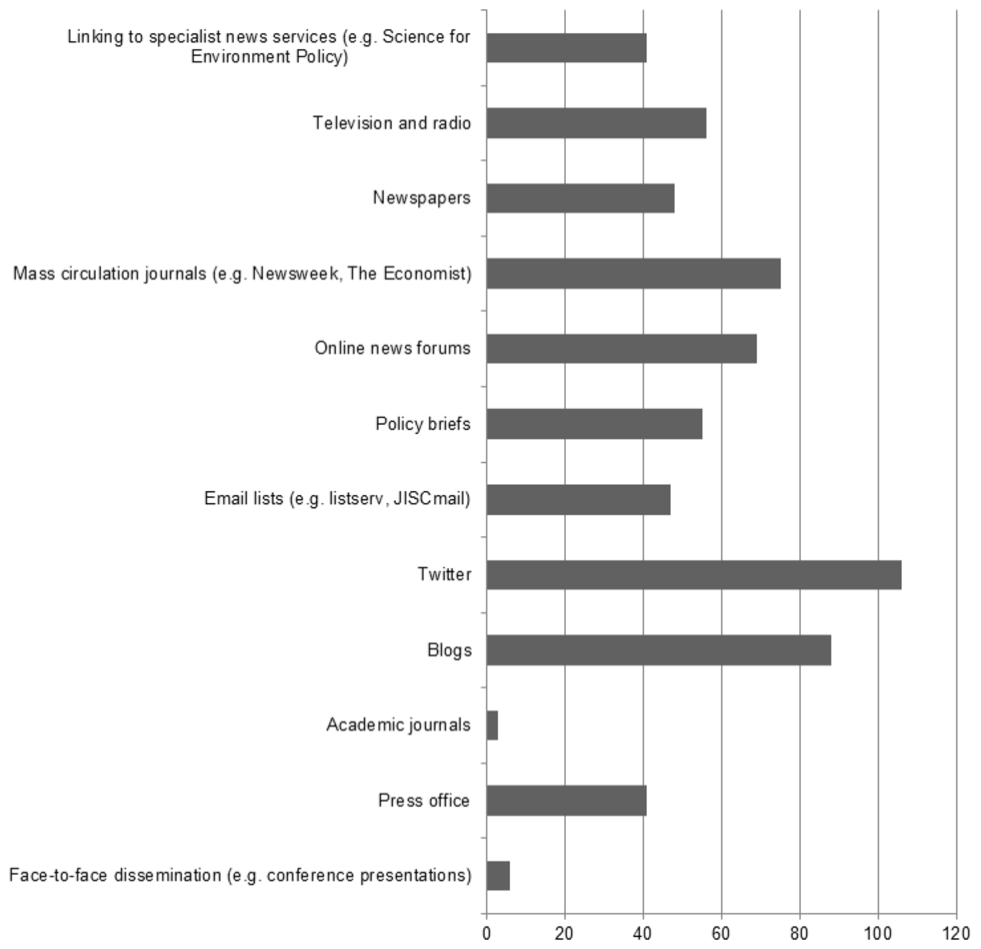
Dissemination Routes to Academic Audiences that are ‘Never Used’ by number.

We were also interested to explore how researchers were disseminating their research to academic and non-academic audiences and whether there had been any changes in this, from their perspective, over the last five years. It is perhaps useful to start from the context of those dissemination routes with which researchers were not particularly engaging. In 2012, many researchers were not using social media to reach academic audiences (Figure 2). In particular, researchers report that they do not use Twitter (n=106, 79%), Blogs (n=88, 65%) or mass media (n=75, 56%) to promote their research to other academics. This is perhaps not surprising, given that specialist media (e.g. academic journals) exist specifically to reach this group. 

In relation to academic audiences, researchers perceived that they had increased the use of academic journal publication (n=70, 51%), face-to-face dissemination (such as conference presentations) (n=44, 32%) and linking to specialist news services, like SfEP (n=37, 27%). This likely reflects that peer-reviewed journal and conference publications remain a mainstay of academic dissemination, but additionally the likelihood that dissemination via such routes continues to increase as a researcher advances in their academic career. Therefore the perceived increase in the use of these tools may be reflective of changes in a researcher’s prominence over time, rather than variation in their value within the academic community. Use of specialist news services is also reflective of the route which was used to survey researchers, and their awareness of such opportunities following engagement with SfEP. 

With regards to non-academic audiences we were also interested to examine any possible changes in dissemination, particularly in the context of an increase in interest in this type of activity from external agents (e.g. funders) and general discourses around encouraging researchers to engage with broader publics. It is notable that slightly fewer respondents answered this series of questions, with 132 completing responses, 89% of the sample. Again it is useful to look initially at the sources which were not in popular use. For the majority of researchers, there has been little change in the use of media to communicate with non-academic audiences over the past five years. As Table 3 indicates social media are rarely used as a means for communicating with non-academics, 73% (n=97) never used Twitter, 64% (n=84) never used blogs and 51% (n=67) never use online news forums. When comparing the use of these media as tools to communicate with academic and non-academic audiences, it is apparent that the majority of researchers do not find these to be suitable tools for disseminating their research. Those that were not using social media to communicate with academic audiences were also not using them to communicate with non-academic audiences. In addition, it was notable that a large number of respondents did not use mainstream media such as television and radio (n=51, 39%) or newspapers (n=44, 30%) to disseminate their research. 

**Table 3:** Dissemination Routes to Non- Academic Audiences.

**Routes to Dissemination for Non-Academic Audiences**	**Use more**	**Use about the same**	**Use less**	**Never use**	**Total N**
Face-to-face dissemination (e.g. speaking at a science festival)	36	63	7	26	132
Press office	27	55	8	42	132
Academic journals	37	74	4	17	132
Blogs	17	26	5	84	132
Twitter	12	19	4	97	132
Email lists (e.g. listserv, JISCmail)	23	41	11	57	132
Policy briefs	21	52	10	49	132
Online news forums	16	41	8	67	132
Mass circulation journals (e.g. Newsweek, The Economist)	9	45	8	70	132
Newspapers	22	51	15	44	132
Television and radio	26	44	11	51	132
Linking to specialist news services (e.g. Science for Environment Policy)	33	51	6	42	132

Face-to-face communication is used by a majority of respondents and around 27% (n=36) indicate an increase in face-to-face contact with non-specialist audiences. Combining the categories of ‘increasing use’, and ‘about the same’ it is clear that face-to-face communication is one of the most likely means for non-academic dissemination, at 74% (n=99). Similarly when combining these two response categories, a majority of respondents make use of their press office for dissemination (62%, n=82). As the press office most likely produces a media release aimed at mass media (newspapers, TV, radio and other print media), it is worth noting that this figure is still consistent with a large minority of researchers who never use mass media to communicate with non-academic audiences (e.g. 39% who do not use TV or radio and 30% who do not use newspapers). Furthermore, what these data do not tell us is how frequent these interactions are, and that is also worthy of further exploration. 

### Outcomes of research dissemination

Within the questionnaire we took the opportunity to ask researchers about their views towards the potential outcomes of their work featuring in news services like SfEP. Here a number of possible outcomes were viewed as ‘very’ or ‘somewhat’ positively by researchers (Table 4). It should be again noted that the sampling method may have positively influenced responses to this question, but the connection to the service provided a tangible example to explore the aspirations and reach of this small group of researchers. 

**Table 4:** Views towards outcomes of dissemination.

**Outcomes**	**Very positive/somewhat positive**
Create links between scientists and people working in business and industry	100 (72%)
Help my research to reach policy-makers	116 (83%)
Enable members of the public to learn about my research	111 (80%)
Increase the academic impact of my research	104 (75%)
Help me obtain funding	48 (34%)
Bring my research to the attention of people in important organisations	118 (85%)
Help my research reach an audience beyond my home country / region	115 (83%)
Provide a route for access to my original publications (where this is possible)	107 (77%)
Increase the number of irrelevant emails that I receive	20 (14%)
Make it more likely that I will be contacted by lobbyists	44 (32%)
Open my research to criticism from members of the public / other scientists	91 (65%)
Open my research to criticism from policy-makers	90 (65%)
Increase my personal profile as a researcher	108 (78%)

Researchers were most positive about the possibility of news services such as SfEP bringing their research to the attention of people in important organisations (n=118, 85%). However news services such as SfEP also offer the potential to reach beyond regional and national borders and these were seen to be favourable outcomes for 83% (n=115) of the researchers responding to these questions. 83% (n=116) were also positive around it helping their research to reach policymakers, closely followed by 80% (n=111) of respondents who were positive towards it enabling members of the public to learn about their research and it providing a route to original publications. 

In general researchers were positive about services such as SfEP disseminating their research to policymakers, organisations and the public despite the recognition amongst large numbers that this could also open themselves up for criticism, approximately 65% (n=90 to 91) of researchers responded that it could open their research to criticism from policymakers or members of the public and other scientists. Finally, it was notable that very few researchers made a connection between such opportunities for dissemination and research funding. Just 34% (n=48) responded positively that having their research featured in a news service like SfEP could help them to obtain funding, suggesting that very few researchers are making any connection between the profile of their work, its impact and the potential for future funding and support. 

## Discussion and Conclusions

The results of this survey of a relatively small number of researchers involved in environmental science point to some interesting trends and approaches to dissemination, both to academic and non-academic communities. 

Despite calls for broader communication of scientific activities it is clear that publishing in academic journals and presenting at academic conferences remain the gold standard and primary dissemination route for many researchers. Calls for research to be published in open access formats (whether in open access journals or via institutional research repositories) [20,21] fit nicely into this discourse, enabling researchers to do what they already do. Open access formats and dissemination opportunities such as SfEP allow researchers to utilise the opportunities of the internet for dissemination, whilst adhering to principles of peer-reviewed, good quality research [22].

However, it is predicated on an assumption that non-academic publics can and will access research in such places, and that researchers will use dissemination opportunities that are of convenience to them. In essence, public communication is seen as being achieved by providing a ‘right of entry’ alone. However, we would argue that greater access must also be coupled with complimentary materials that recognise that non-academic audiences may have different expectations of, interactions with and uses for research. Providing a ‘right of entry’ seems unlikely to meet these needs and expectations, mediating, translating and tailoring research material to enable it to be used or assimilated by non-academic audiences is a key role within science communication [23]. Social media is ‘today’s reality’ for science communication but there are all sorts of issues in how it influences traditional media environments, filters information and influences societal debates which are only just beginning to be understood [24]. 

It is notable that while a significant minority of respondents never use a press office as a means of disseminating their research, a majority of researchers recognise their press office as a route to reaching non-academic publics. Press offices are skilled at translating research into stories that appeal to journalists, and as such they contribute to what has been termed an ‘information subsidy’ given to the journalism industry. This raises questions about the credibility of journalism in an era where news stories are influenced by institutional agendas and journalism is increasingly referred to as churnalism [12]. As Berkowitz [25] (p. 81) notes, “put most simply, news sources exert a stronger influence over the news agenda than do journalists.” Nevertheless, from the perspective of researchers, it makes sense to draw upon the specialist skills of press officers to maximise the chances of their research being covered in mass media. What is perhaps more surprising is the relatively large minority that do not make use of this service. Khot [13] notes that this may simply be a case of researchers forgetting that this service is available and that both press officers and researchers highlight lack of time as a factor that restricts their ability to work together. Our findings would support the notion that researchers remain relatively non-strategic in their dissemination strategies. 

A key outcome, which is perhaps under-recognised in terms of dissemination strategies, is the opportunity dissemination brings for the creation of contacts. Coverage in the news service, traditional and social media all resulted in communication for some researchers with other interested researchers, but key was the opportunity to communicate with policymakers, publics and journalists, which can be almost impossible to establish based on academic journal publication alone. Bultitude et al. [26] note that fostering links between policymakers and researchers is a priority for those seeking to facilitate evidence based policy. While the research community is beginning to recognise the importance of communicating with policymakers and publics and to reach out to these communities [2,27] the questions asked of researchers in this study around how non-academic audiences are communicated with suggest many still struggle to interact publicly.

A number of responses suggest that researchers are still adapting to more novel communication approaches (such as the potential of social media) and are engaging largely for individual reasons, rather than to fulfil a particular communication or impact agenda. Very few researchers were utilising non-traditional approaches to communicate either with other researchers or non-academics. These findings tend to support Trench’s [17] contention that the internet is not living up to its potential as a space where interested individuals and scientists can exchange views, though whether the barrier lies in the technology or within the culture of scientists remains to be explored. Although preliminary, it is interesting to note the emergence of different approaches to engaging with traditional and social media apparent in the different regions of Europe, with Northern European countries more likely to use traditional media and variation in engaging with social media. This may reflect differences in cultural and communicative preferences and is worth further exploration. 

Those that were using social media reported fruitful outcomes from such communication, particularly increased awareness amongst the journalistic community. This perhaps suggests that journalistic communities are making more use of social media resources than either policymakers or the public. Equally it could reflect the differing ways in which social media are used. Its transient nature means social media allows for direct contact at the time of posting. For journalists, this means that social media can broker contact with experts either to initiate a new story or to add depth to a story which is already on the news agenda. However, it also means that social media may not be seen as useful for those exploring research over a longer timescale, for example through retrospective searching. Though these results are tentative at this stage, based on the lower numbers of respondents involved, they do suggest that further examination of the potential of social media to disseminate research to both academic and non-academic audiences is warranted. 

Peters [2] notes that researchers tend to view media interactions as having a positive, neutral or balanced effect (but hardly ever negative impact) on their careers. Yet in our survey, few researchers made a link between efforts to disseminate their research to either academic or non-academic audiences and outcomes of funding applications (which might be considered important for their careers). This suggests that although funding agencies are now encouraging the embedding of impacts, including public engagement, within research, few researchers are making this connection to opportunities for engaging in a wider range of dissemination activities for a wider range of audiences. By better recognising the value of these activities at an individual level and beyond institutional agendas, researchers might be enabled to highlight the impact of their research in future funding applications as well as demonstrating wider impacts of their research. 

## Conclusion

Taken together, the results suggest that researchers are reacting to opportunities for dissemination rather than developing particular strategies to communicate with or reach specific audiences and very few researchers are actively using social media to disseminate their research findings. It seems likely that many dissemination activities that do not employ the traditional routes of academic journals and conference presentations may be serendipitous rather than strategic or planned. This outcome is not surprising; it replicates prior findings in the literature which suggest researchers often lack awareness of the role of the media in disseminating research. This combined with a sense that they are time poor means proactive communication is not prioritised. However it is nevertheless something which could be better considered in the design of communication approaches for scientists (for example, are there quick, effective techniques that can be embedded for scientists to further share, disseminate, capture, record their researches impact?). Equally, communication training and resources for researchers could better support them to plan, devise and support communication approaches in creative ways. 
